# The grit personality trait, eating behavior, and obesity among Japanese adults: a cross-sectional study

**DOI:** 10.1186/s13030-025-00337-9

**Published:** 2025-08-22

**Authors:** Noriaki Kurita, Takako Maeshibu, Tetsuro Aita, Takafumi Wakita, Hiroe Kikuchi

**Affiliations:** 1 Department of Clinical Epidemiology, Graduate School of Medicine, Fukushima Medical University, 1 Hikarigaoka, Fukushima City, 960-1295 Fukushima Japan; 2 Department of Innovative Research and Education for Clinicians and Trainees (DiRECT), Fukushima Medical University Hospital, Fukushima, Japan; 3 Division of Rheumatology, Department of Medicine, Showa Medical University School of Medicine, Tokyo, Japan; 4 Department of Research, Patient Driven Academic League (PeDAL), Tokyo, Japan; 5 Graduate School of Psychology, Kansai University, Osaka, Japan; 6 Department of General Internal Medicine and Family Medicine, Fukushima Medical University, Fukushima, Japan; 7 Department of Sociology, Kansai University, Osaka, Japan; 8 Department of Psychosomatic Medicine, National Center for Global Health and Medicine, Japan Institute for Health Security, Tokyo, Japan

**Keywords:** Obesity, Grit, Three-factor eating questionnaire-R21, Eating behavior, Mediation analysis, Stigma

## Abstract

**Background:**

Obesity is a chronic disease influenced by genetic, cultural, environmental, and psychosocial factors, making it difficult to manage through individual effort alone. Despite this complexity, obesity is often attributed to a lack of willpower and poor control over eating behaviors, contributing to stigma. However, research on this issue remains limited. This study quantified the extent to which multidimensional eating behaviors statistically explained the association between obesity and grit, which shared characteristics with self-control.

**Methods:**

We conducted a cross-sectional study involving Japanese adults across a wide range of age groups. Grit was measured using the 8-item Short Grit Scale. Multidimensional eating behaviors were measured using the Japanese version of the 21-item Three-Factor Eating Questionnaire-R21, including uncontrolled eating, emotional eating, and cognitive restraint. Obesity was defined as a body mass index ≥ 25.0 kg/m^2^. A series of logistic regression models were created to analyze the association between grit and obesity with and without eating behaviors. Mediation analyses using the Karlson-Holm-Breen method were performed.

**Results:**

Of the 1,641 adults, 26.8% had obesity. Higher grit level was associated with a lower likelihood of obesity, less uncontrolled and emotional eating, and higher cognitive restraint. Grit was positively associated with cognitive restraint and negatively associated with uncontrolled and emotional eating; these multidimensional eating behaviors statistically accounted for the association between grit and obesity. Uncontrolled and emotional eating fully accounted for the association, whereas cognitive restraint partially accounted for it. These findings are consistent with the possibility of mediation through eating behavior in the relation between grit and obesity.

**Conclusions:**

Our findings suggest that healthcare providers and policymakers should prioritize addressing multidimensional eating behaviors that explain the link between grit and obesity rather than on grit itself. Identifying and managing impairments in eating behavior rather than attributing obesity to an individual’s lack of willpower may help reduce stigma and support effective obesity prevention strategies.

**Supplementary Information:**

The online version contains supplementary material available at 10.1186/s13030-025-00337-9.

## Background

Obesity is a chronic disease that increases the risk of cardiovascular disease [1], and its prevalence continues to increase globally. From 1975 to 2014, the worldwide mean body mass index (BMI) increased by 2.5 kg/m² in men and 2.3 kg/m² in women [2]. Japan showed a similar trend, particularly among men [3, 4]. Obesity is influenced by a complex interplay of genetic predisposition, cultural background, environmental factors, and psychosocial contexts, making it difficult to manage through individual effort alone. Nevertheless, obesity is often attributed to a lack of willpower or poor self-control over eating behavior, contributing to widespread stigma. Such negative stereotypes are not only perpetuated by the general public, but also by healthcare professionals, employers, and policymakers [5]. Rather than providing support, these prejudices may impede access to appropriate care for individuals with obesity [5]. Sustained behavioral changes and effective motivational support are essential for preventing obesity-related complications and improve the well-being of people with obesity [6, 7]. Thus, it is important to assess multiple aspects of eating behavior, including physiological sensations, restrained eating, and emotional eating [6]. However, despite the persistence of stigma associated with obesity, there is limited research exploring how personality traits such as grit, which shares characteristics with willpower, and multidimensional eating behaviors interact to influence obesity.

Grit, defined as the ability to maintain a commitment to long-term goals despite challenges [8], is associated with healthy eating behaviors [9] and a low prevalence of obesity [10, 11]. Indeed, evidence suggests that individuals with low grit tend to prefer immediate pleasurable behaviors, such as choosing dessert first [12, 13]. However, several cross-sectional studies from the United States that reported associations between higher grit and lower BMI or lower odds of being overweight did not account for differences in exercise habits and eating behaviors [10, 11]. Another study linking grit to regular eating and healthy food choices did not address eating behaviors related to negative emotions or hunger [9]. Multidimensional eating behaviors, such as emotional eating (EE; overeating in response to negative emotions), uncontrolled eating (UE; general difficulties in regulating eating, including hunger), and cognitive restraint (CR; conscious restriction of food intake to manage weight), can be assessed using the Three-Factor Eating Questionnaire (TFEQ) [14, 15]. However, only one study that examined grit in relation to eating behaviors using the TFEQ measured CR alone, omitting EE and UE [16]. More importantly, to date, no studies have explored the extent to which these multidimensional eating behaviors mediate the relation between grit and obesity.

Therefore, this study aimed to examine the interrelations among grit, multidimensional eating behaviors, and obesity in a diverse sample of Japanese adults. Furthermore, we quantified the extent to which multidimensional eating behaviors mediate the association between grit and obesity. If this association is largely mediated by eating behavior, it would support healthcare providers and policymakers in shifting their focus toward assessing individual eating patterns and developing tailored interventions or coordinated policies rather than unjustly attributing obesity to a lack of willpower.

## Methods

### Aim, design, and setting

This study aimed to examine the interrelations between grit, multidimensional eating behaviors, and obesity in a wide range of adult Japanese patients of both sexes. This cross-sectional, online survey was approved by our institutional review board (ippan2022-210) and conducted with the assistance of a web-based research company (Cross Marketing, Tokyo, Japan). To ensure balanced representation, participants (*N* = 1,500) were recruited using stratified sampling by sex (1:1), age (≥ 65 vs. <65), and obesity-related status (see Additional File 1 for details). Obesity-related stratification was based on self-reported experience with obesity (treatment history, concern about obesity, or neither), using predefined panel categories. Participants were offered incentive points that could be redeemed for cash, gift certificates, or mileage. Participants answered an online questionnaire prepared by the company. The response data were collected between January 26 and 31, 2023, and stored on the company server. The participants were instructed to respond only once. Only those who provided informed consent completed the questionnaire.

### Screening items

To minimize the inclusion of careless participants [17], we applied five exclusion criteria: inappropriate entries for (1) age or sex, (2) extreme values for height or weight, and (3) completion time < 5 min [18, 19] (Additional File 1). No missing data were present, as all items were mandatory in the online survey. We did not define or exclude responses based on inconsistency or careless patterns within scales; all submitted responses were treated as valid. Other than excluding participants with extreme values for height or weight, no additional exclusion criteria based on extreme BMI values were applied in this study. We assessed consistency by asking respondents to report their age and sex twice (at the beginning and end of the survey); those with mismatched responses were excluded. Height and weight were asked only once.

### Grit

The exposure in this study was the grit personality trait assessed using the Japanese version of the eight-item Short Grit Scale [8, 20]. The concept of grit comprises two components: a passion for and perseverance toward long-term goals [21]. These components are typically defined as “consistency of interest”, which refers to maintaining stable goals and sustained interest over time (e.g., staying focused on a project lasting several months or more), and “perseverance of effort”, which reflects continued effort and resilience despite setbacks (e.g., not being discouraged by failure and persisting until completion). Respondents were instructed to rate each item on a Likert scale ranging from 1 (“Not at all like me”) to 5 (“Very much like me”). The scores for the four negatively worded items were reversed, and the overall score was calculated as the average of all items, ranging from a minimum of 1 to a maximum of 5. The alpha coefficient for the Short Grit Scale was 0.74. The construct validity of the scale was verified using confirmatory factor analysis [20].

### Obesity

The primary outcome was obesity, defined as a BMI ≥ 25.0 kg/m^2^. This threshold, recommended by the World Health Organization for Asian populations in the Asia-Pacific region [22], is also endorsed by the Japan Society for the Study of Obesity [23]. It reflects the level of risk of diabetes mellitus and hypertension in Japanese and other Asian populations [23]. The BMI was calculated using self-reported weight and height (kg/m²).

### Eating behavior: the three-factor eating questionnaire-R21

The mediator in this study, multidimensional eating behavior, was measured using the Japanese version of the 21-item Three-Factor Eating Questionnaire-R21 (TFEQ-R21), developed by Cappelleri and Karlsson [24]. With permission from the original developer (Karlsson), the scale was translated into Japanese by two physicians experienced in scale development. It was then back-translated into English by two bilingual translators (one American and one Canadian), and necessary revisions were made to the translated version by comparing the wordings with the original. Finally, the back-translated and translated versions were sent to the original author, and the final version was approved (see Additional File 2).

Participants were instructed as follows: “This section contains statements and questions about eating behaviors and the feeling of hunger. Read each statement carefully and select the option that best applies to you.” Items 1–20 are rated on a 4-point Likert scale, and item 21 on an 8-point numerical rating scale. Responses were recoded before analysis: items 1–16 were reverse coded; item 21 was recoded into four categories (1–2 = 1, 3–4 = 2, 5–6 = 3, 7–8 = 4) [24]. Domain scores were then calculated as a transformed score ranging from 0 to 100, where the sum of all items was subtracted from the lowest possible raw score, divided by the range of possible raw scores, and multiplied by 100. Higher scores for CR (6 items), UE (9 items), and EE (6 items) indicated greater CR, UE, and EE.

### Other survey variables

Item selection details are provided in Additional File 3. The Japanese version of the 33-item Dutch Eating Behavior Questionnaire (DEBQ) includes three domains: emotional eating, external eating, and restrained eating [25, 26]. Items were rated on a 5-point Likert scale (1 = never to 5 = very often). Domain scores were calculated as the mean of relevant items [26]. Higher scores indicate a stronger tendency toward each behavior. The alpha coefficients for each domain were as follows: emotional eating, 0.95; external eating, 0.73; and restrained eating, 0.87 [25].

Demographic characteristics (age, sex, education level, total household income, and marital status), health behaviors (exercise habits, smoking history, and alcohol consumption), and non-communicable diseases (NCDs) were included as covariates. To assess NCDs, participants were asked the following question: “Have you ever been told by a physician that you have any of the following diseases?” They then selected one of the following nine conditions: diabetes mellitus, cancer, kidney disease, stroke, congestive heart failure, chronic lung disease, eating disorders, depression, or other psychiatric disorders. For each condition, three response options were provided: (1) Never told, (2) Told in the past and no longer visiting a physician, (3) Told and currently visiting a physician. Responses (2) and (3) indicate the presence of the condition. Information regarding the use of medications for these conditions was not collected. Regarding eating disorders, we did not specify diagnostic subtypes in the questionnaire; thus, it was assumed that respondents with binge eating disorder would select the item for “eating disorders.”

### Statistical analyses

Psychometric analyses were performed using R (version 4.1.2), with the psych (v2.2.3) and lavaan (v0.6-11) packages. All other analyses were performed using Stata/SE version 17 (Stata Corp., College Station, TX, USA). Respondent characteristics were summarized as means and standard deviations (SDs) or medians with interquartile ranges for continuous variables, and as frequencies and proportions for categorical variables.

To evaluate the TFEQ-R21, three-factor confirmatory factor analysis was done using the recoded raw scores (items 1–16 and item 21). Model fit was assessed using the comparative fit index (CFI ≥ 0.90) and root mean square error of approximation (RMSEA ≤ 0.08) [27]. Acceptable standardized loadings were set at ≥ 0.3 [28]. We also examined item-level distributions to identify floor or ceiling effects, defined as > 50% of responses at the extremes [24]. The internal consistency reliability of each domain was assessed by Cronbach’s α and McDonald’s ω coefficients [29]. Construct validity was examined via correlations between TFEQ-R21 and DEBQ domain scores, and criterion validity was assessed by correlating TFEQ-R21 scores with BMI using Pearson correlation coefficients. Hypothesized relations are detailed in Additional File 4.

**Fig. 1:**
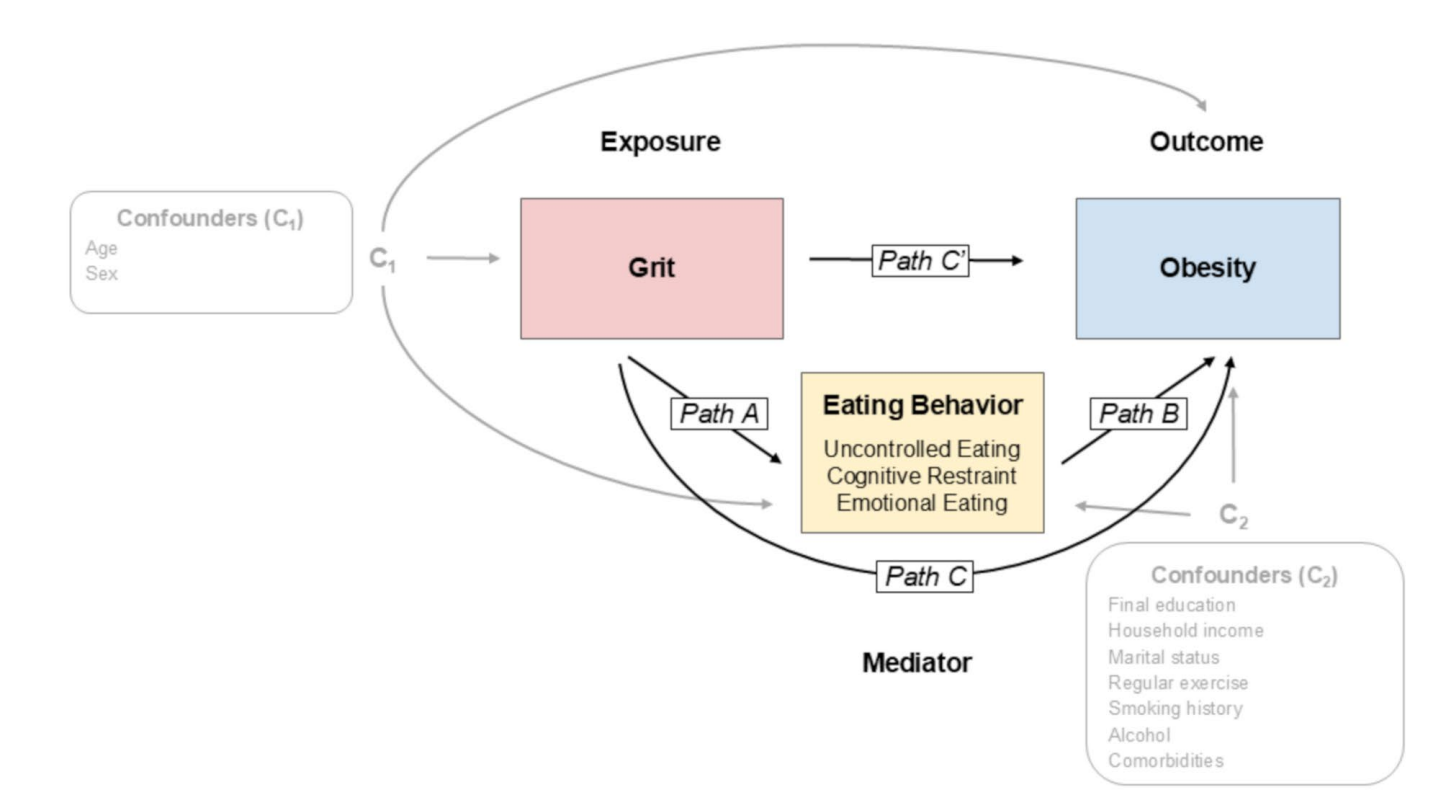
Analytic framework. Path C represents the total effect of grit on obesity. Paths A and B represent the indirect effect mediated by eating behaviors. Path C represents the direct effect of grit on obesity-controlled eating behaviors

Mediation analyses were conducted to examine whether multidimensional eating behavior variables mediated the association between grit and obesity, based on the conceptual framework shown in Fig. 1. First, a series of regression models were fitted to informally assess the mediators by observing relative changes in the magnitude of the parameters, capturing the association between the exposure variable (i.e., grit) and obesity. Specifically, the association between grit and eating behavior was analyzed using general linear models adjusted for the covariates (see Fig. 1, path A), and the association between grit and obesity with and without eating behavior was analyzed using logistic regression models adjusted for the covariates (without eating behavior: Fig. 1, path C; with eating behavior: paths C’ and B). While we reported model fit indices (e.g., pseudo R² for logistic regression models, adjusted R² for general linear models), these were for auxiliary purposes, as our primary focus was on estimating associations rather than predictive performance [30]. Subsequently, formal mediation analyses were conducted using the Karlson–Holm–Breen (KHB) method in Stata [31], which decomposes effects in nonlinear probability models such as logistic regression models. This approach allowed estimation of total, direct (grit on obesity), and indirect (via eating behavior) effects, as well as the proportion mediated. The association between grit and obesity was considered fully mediated if the association became nonsignificant after adjustment for eating behavior, and partially mediated if the association persisted [32]. The aforementioned analyses were performed separately for each TFEQ-R21 domain, UE, CR, and EE, partly due to a strong correlation between some domains, as indicated by Pearson correlation coefficients (see Results). Sensitivity analyses further decomposed the total effect of grit into direct and multiple indirect pathways, additionally treating exercise habits, smoking, and alcohol consumption as mediators using the KHB method [31]. Statistical significance was set at *P* < 0.05 for all analyses.

## Results

**Fig. 2:**
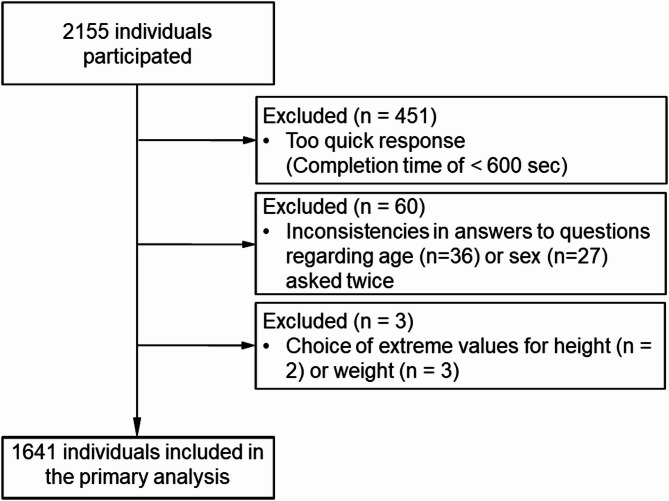
Flow of the study

Of 2,155 participants, 514 were excluded due to careless responses or extreme values, leaving the data of 1,641 individuals available for the primary analysis (Fig. 2).

### Participant characteristics

The mean age was 60.6 years, and 786 (47.9%) were male (Table 1). Diabetes mellitus was the most common comorbidity (18%), followed by depression (12.5%) and malignant diseases (11.5%). Regular exercise was reported by 31.0%, daily alcohol use by 19.8%, and smoking by 18.1%. The mean ± SD (5th − 95th percentile) BMI was 23.0 ± 4.7 (17.2–31.8) kg/m^2^; in addition, 439 (26.8%) participants had obesity.

**Table 1:** Participant characteristics by obesity status (*N* = 1641)

	Obesity		Total
	*No*	*Yes*	
	*n* = 1202	*n* = 439	*N* = 1641
*Demographics*			
Age, y	61.1 (13.1)	59.2 (12.0)	60.6 (12.9)
Male, n (%)	549 (45.7%)	237 (54.0%)	786 (47.9%)
Education, n (%)			
Junior high school	31 (2.6%)	18 (4.1%)	49 (3.0%)
High school	378 (31.5%)	161 (36.7%)	539 (32.9%)
Professional Training College / College of Technology / Junior College	270 (22.5%)	82 (18.7%)	352 (21.5%)
University	481 (40.0%)	160 (36.5%)	641 (39.1%)
Graduate school	42 (3.5%)	18 (4.1%)	60 (3.7%)
Household income, n (%)			
< 1 000 000 yen	97 (8.1%)	41 (9.3%)	138 (8.4%)
1 000 000–4 999 999 yen	660 (54.9%)	242 (55.1%)	902 (55.0%)
5 000 000–9 999 999 yen	326 (27.1%)	123 (28.0%)	449 (27.4%)
≥ 10 000 000 yen	119 (9.9%)	33 (7.5%)	152 (9.3%)
Marital status, n (%)			
Unmarried	227 (18.9%)	110 (25.1%)	337 (20.5%)
Married	791 (65.8%)	258 (58.8%)	1049 (63.9%)
Divorced	116 (9.7%)	48 (10.9%)	164 (10.0%)
Widowed	68 (5.7%)	23 (5.2%)	91 (5.6%)
*Comorbidities*			
Diabetes mellitus, n (%)	139 (11.6%)	157 (35.8%)	296 (18.0%)
Malignancy, n (%)	139 (11.6%)	50 (11.4%)	189 (11.5%)
Renal disease, n (%)	46 (3.8%)	32 (7.3%)	78 (4.8%)
Stroke, n (%)	24 (2.0%)	14 (3.2%)	38 (2.3%)
Congestive heart failure, n (%)	7 (0.6%)	5 (1.1%)	12 (0.7%)
Chronic lung disease, n (%)	21 (1.8%)	8 (1.8%)	29 (1.8%)
Eating disorder, n (%)	24 (2.0%)	16 (3.6%)	40 (2.4%)
Depression, n (%)	96 (8.0%)	109 (24.8%)	205 (12.5%)
Other mental disorders, n (%)	74 (6.2%)	82 (18.7%)	156 (9.5%)
*Psycho-behavioral characteristics*			
Regular exercise, n (%)	417 (34.7%)	104 (23.7%)	521 (31.8%)
Smoking, n (%)	212 (17.6%)	85 (19.4%)	297 (18.1%)
Alcohol consumption, n (%)			
Rarely or never (including inability to drink)	593 (49.3%)	263 (59.9%)	856 (52.2%)
Sometimes	366 (30.5%)	94 (21.4%)	460 (28.0%)
Every day	243 (20.2%)	82 (18.7%)	325 (19.8%)
Grit-S, points	3.1 [2.9,3.5]	3.0 [2.6,3.4]	3.1 [2.8,3.5]
Cognitive restraint, points	38.9 [22.2,50]	44.4 [33.3,55.6]	38.9 [22.2,50]
Uncontrolled eating, points	25.9 [11.1,37.0]	37.0 [25.9,51.9]	29.6 [14.8,40.7]
Emotional eating, points	11.1 [0,33.3]	33.3 [11.1,44.4]	16.7 [0,33.3]

### Eating behavior scale (TFEQ-R21): descriptive and psychometric properties

The model demonstrated acceptable fit (RMSEA = 0.069, CFI = 0.924; Additional File 5), and reliability was good across domains (Cronbach’s alpha coefficients: 0.79 for CR, 0.89 for UE, and 0.92 for EE). The lowest standardized loading (item 17: 0.31) was acceptable considering the aforementioned model fit and reliability (Additional File 6).

The mean ± SD for the CR, UE, and EE domain scores were 38.5 ± 20.0, 30.1 ± 19.3, and 22.4 ± 22.2, respectively. At the item level, three items comprising the EE domain had the lowest percentage scores of > 50%; however, no item-level floor effect was observed in the obesity population (Additional File 7).

The UE domain showed moderate correlations with DEBQ “emotional eating” and “external eating,” but weak correlation with “restrained eating.” (Additional File 8). The CR domain was strongly correlated with “restrained eating” but showed weak or negligible correlation with the other DEBQ domains. The EE domain correlated strongly with DEBQ “emotional eating,” moderately with “external eating,” and weakly with “restrained eating.” All TFEQ-R21 domains were weakly, positively correlated with BMI (Additional File 8). These results support the construct validity of the TFEQ-R21. A strong correlation was observed between the UE and EE domains (Additional File 9).

### Association between grit and eating behavior

The median grit score was 3.1 (interquartile range [IQR]: 2.8–3.5). As shown in Table 2, higher grit was associated with lower UE (per 1-point increase: −7.63 [95% confidence interval (CI): −9.13, − 6.12]) and EE (per 1-point increase: −6.98 [95% CI: -8.73, -5.23]), and higher CR (per 1-point increase: 2.51 [95% CI: 0.85, 4.18]). UE was positively associated with smoking, diabetes, kidney disease, eating disorders, and depression; and negatively with age, male sex, and chronic lung disease. EE was positively associated with bereavement, eating disorders, depression, and other psychiatric conditions; and negatively with age, male sex, and chronic lung disease. CR was positively associated with diabetes and depression; and negatively with smoking.

**Table 2:** Associations of eating behavior with grit personality and covariates^1^ (*N* = 1641)

	Uncontrolled eating			Cognitive restraint			Emotional eating	
	Mean difference, point estimate (95% CI)	*P*		Mean difference, point estimate (95% CI)	*P*		Mean difference, point estimate (95% CI)	*P*
**Grit-S**,** per 1-point increase**	**-7.63 (-9.13 to -6.12)**	**< 0.001**		**2.51 (0.85 to 4.18)**	**0.003**		**-6.98 (-8.73 to -5.23)**	**< 0.001**
Age, per 10-yr increase	**-3.09 (-3.92 to -2.26)**	**< 0.001**		-0.81 (-1.72 to 0.11)	0.084		**-2.77 (-3.73 to -1.81)**	**< 0.001**
Male vs. female	**-3.09 (-5.06 to -1.13)**	**0.002**		-2.05 (-4.22 to 0.12)	0.064		**-5.10 (-7.38 to -2.82)**	**< 0.001**
Education								
Junior high school	Reference			Reference			Reference	
High school	1.15 (-4.11 to 6.41)	0.669		-0.36 (-6.18 to 5.46)	0.903		2.55 (-3.56 to 8.66)	0.413
Professional Training College / College of Technology / Junior College	1.04 (-4.37 to 6.45)	0.706		-0.73 (-6.71 to 5.26)	0.812		1.33 (-4.95 to 7.61)	0.679
University	2.2 (-3.11 to 7.51)	0.417		0.38 (-5.49 to 6.25)	0.899		4.36 (-1.80 to 10.52)	0.165
Graduate school	1.26 (-5.62 to 8.13)	0.720		-1.75 (-9.35 to 5.85)	0.652		3.34 (-4.64 to 11.32)	0.412
Household income								
< 1 000 000 yen	Reference			Reference			Reference	
1 000 000–4 999 999 yen	-1.11 (-4.35 to 2.14)	0.504		1.15 (-2.44 to 4.75)	0.529		0.64 (-3.13 to 4.41)	0.741
5 000 000–9 999 999 yen	-0.03 (-3.57 to 3.51)	0.987		1.90 (-2.01 to 5.82)	0.341		0.58 (-3.53 to 4.68)	0.783
≥ 10 000 000 yen	0.80 (-3.54 to 5.13)	0.718		**5.04 (0.24 to 9.84)**	**0.039**		1.99 (-3.05 to 7.02)	0.439
Marital status								
Unmarried	Reference			Reference			Reference	
Married	1.41 (-1.08 to 3.91)	0.266		0.62 (-2.13 to 3.38)	0.657		0.70 (-2.19 to 3.60)	0.633
Divorced	0.13 (-3.33 to 3.58)	0.942		1.66 (-2.16 to 5.48)	0.393		-0.08 (-4.09 to 3.93)	0.967
Widowed	3.89 (-0.57 to 8.36)	0.087		2.25 (-2.68 to 7.19)	0.371		**6.81 (1.63 to 11.99)**	**0.010**
Regular exercise, yes	1.10 (-0.80 to 3.00)	0.255		1.69 (-0.41 to 3.78)	0.115		0.21 (-1.99 to 2.42)	0.850
Smoking, yes	**2.55 (0.20 to 4.89)**	**0.033**		**-3.61 (-6.20 to -1.01)**	**0.006**		1.08 (-1.65 to 3.80)	0.439
Alcohol consumption								
Rarely or never (including inability to drink)	Reference			Reference			Reference	
Sometimes	0.22 (-1.83 to 2.26)	0.837		1.99 (-0.28 to 4.25)	0.086		0.61 (-1.77 to 2.98)	0.618
Every day	-2.11 (-4.53 to 0.31)	0.088		-1.29 (-3.97 to 1.39)	0.345		-2.25 (-5.06 to 0.56)	0.116
Reported comorbidities								
Diabetes mellitus	**7.70 (5.34 to 10.06)**	**< 0.001**		**7.76 (5.15 to 10.37)**	**< 0.001**		**8.06 (5.31 to 10.80)**	**< 0.001**
Malignancy	2.68 (-0.13 to 5.48)	0.061		1.99 (-1.11 to 5.09)	0.209		3.82 (0.57 to 7.08)	0.021
Renal disease	**4.40 (0.02 to 8.77)**	**0.049**		-2.09 (-6.93 to 2.75)	0.397		3.38 (-1.71 to 8.46)	0.193
Stroke	3.71 (-2.56 to 9.98)	0.246		3.60 (-3.33 to 10.54)	0.309		4.21 (-3.07 to 11.49)	0.256
Congestive heart failure	-4.89 (-17.02 to 7.25)	0.430		3.07 (-10.36 to 16.50)	0.654		-1.95 (-16.05 to 12.14)	0.786
Chronic lung disease	**-9.58 (-16.84 to -2.32)**	**0.010**		-6.51 (-14.55 to 1.52)	0.112		**-15.76 (-24.19 to -7.33)**	**< 0.001**
Eating disorder	**9.20 (2.76 to 15.65)**	**0.005**		2.52 (-4.60 to 9.65)	0.488		**12.92 (5.44 to 20.40)**	**0.001**
Depression	**3.79 (0.64 to 6.94)**	**0.018**		**4.43 (0.94 to 7.91)**	**0.013**		**6.50 (2.84 to 10.16)**	**0.001**
Other mental disorder	1.68 (-1.85 to 5.20)	0.351		2.13 (-1.77 to 6.02)	0.285		**4.85 (0.76 to 8.94)**	**0.020**
Adjusted R^2^	0.1631			0.0526			0.1522	

^1^General linear models were fitted with the inclusion of all variables listed above. These three models evaluated Path A, examining the association between grit (exposure) and eating behavior (mediator)

### Association of obesity with grit and eating behavior

Table 3 shows that higher grit was associated with a lower likelihood of obesity (adjusted odds ratio [aOR] per 1-point increase: 0.78 [95% CI: 0.63, 0.97]) in the model without eating behavior. When adjusted for UE or EE, the association between grit and obesity was no longer significant, suggesting full mediation. In contrast, adjusting for CR strengthened the inverse association between grit and obesity (aOR per 1-point increase: 0.73 [95% CI: 0.59, 0.91]). Higher UE, CR, and EE scores were positively associated with obesity (aOR per 10-point increase: 1.39 [95% CI: 1.29, 1.49] for UE; 1.17 [95% CI: 1.1, 1.25] for CR; 1.22 [95% CI: 1.15, 1.29] for EE).

**Table 3:** Associations of obesity with grit personality, eating behavior, and covariates^1^ (*N* = 1641)

	Model without eating behavior			Models with eating behavior							
				Uncontrolled eating			Cognitive restraint			Emotional eating	
	Odds ratio, point estimate (95% CI)	*P*		Odds ratio, point estimate (95% CI)	*P*		Odds ratio, point estimate (95% CI)	*P*		Odds ratio, point estimate (95% CI)	*P*
Grit-S, per 1-pt increase	**0.78 (0.63 to 0.97)**	**0.023**		0.99 (0.79 to 1.24)	0.937		**0.73 (0.59 to 0.91)**	**0.005**		0.88 (0.71 to 1.10)	0.263
**Eating behavior score**,** per 10-pt increase**				**1.39 (1.29 to 1.49)**	**< 0.001**		**1.17 (1.10 to 1.25)**	**< 0.001**		**1.22 (1.15 to 1.29)**	**< 0.001**
Age, per 10-yr increase	0.95 (0.85 to 1.07)	0.401		1.06 (0.94 to 1.20)	0.352		0.96 (0.85 to 1.08)	0.472		1.01 (0.90 to 1.14)	0.875
**Male vs. female**	**2.06 (1.56 to 2.73)**	**< 0.001**		**2.40 (1.79 to 3.22)**	**< 0.001**		**2.18 (1.64 to 2.90)**	**< 0.001**		**2.34 (1.75 to 3.13)**	**< 0.001**
Education											
Junior high school	Reference			Reference							
High school	1.19 (0.60 to 2.35)	0.611		1.16 (0.58 to 2.34)	0.675		1.24 (0.62 to 2.48)	0.536		1.15 (0.58 to 2.30)	0.690
Professional Training College / College of Technology / Junior College	0.86 (0.42 to 1.74)	0.671		0.82 (0.40 to 1.71)	0.602		0.89 (0.44 to 1.83)	0.754		0.84 (0.41 to 1.72)	0.627
University	0.95 (0.48 to 1.89)	0.889		0.87 (0.43 to 1.78)	0.710		0.97 (0.48 to 1.95)	0.923		0.88 (0.43 to 1.77)	0.712
Graduate school	1.00 (0.40 to 2.47)	0.994		0.96 (0.38 to 2.45)	0.940		1.06 (0.42 to 2.66)	0.903		0.95 (0.38 to 2.39)	0.916
Household income											
< 1 000 000 yen	Reference			Reference							
1 000 000–4 999 999 yen	1.23 (0.78 to 1.94)	0.365		1.32 (0.83 to 2.11)	0.247		1.21 (0.77 to 1.91)	0.403		1.22 (0.77 to 1.93)	0.405
5 000 000–9 999 999 yen	1.30 (0.79 to 2.12)	0.300		1.33 (0.80 to 2.21)	0.274		1.28 (0.78 to 2.09)	0.331		1.28 (0.78 to 2.11)	0.333
≥ 10 000 000 yen	1.16 (0.62 to 2.15)	0.639		1.14 (0.60 to 2.15)	0.691		1.09 (0.59 to 2.03)	0.788		1.10 (0.59 to 2.06)	0.769
Marital status											
Unmarried	Reference			Reference							
Married	0.84 (0.60 to 1.18)	0.323		0.78 (0.55 to 1.11)	0.164		0.83 (0.59 to 1.17)	0.280		0.82 (0.58 to 1.16)	0.265
Divorced	0.82 (0.51 to 1.32)	0.417		0.79 (0.49 to 1.29)	0.353		0.78 (0.48 to 1.27)	0.321		0.82 (0.50 to 1.33)	0.413
Widowed	0.91 (0.48 to 1.71)	0.758		0.79 (0.41 to 1.51)	0.476		0.88 (0.46 to 1.66)	0.690		0.8 (0.42 to 1.51)	0.493
**Regular exercise**,** yes**	**0.69 (0.53 to 0.92)**	**0.010**		**0.64 (0.48 to 0.86)**	**0.002**		**0.67 (0.51 to 0.89)**	**0.005**		**0.68 (0.51 to 0.90)**	**0.007**
Smoking, yes	0.88 (0.64 to 1.22)	0.451		0.80 (0.57 to 1.13)	0.205		0.92 (0.67 to 1.28)	0.639		0.86 (0.62 to 1.19)	0.367
**Alcohol consumption**											
Rarely or never (including inability to drink)	Reference			Reference							
**Sometimes**	**0.67 (0.50 to 0.90)**	**0.008**		**0.65 (0.48 to 0.88)**	**0.006**		**0.65 (0.48 to 0.87)**	**0.004**		**0.65 (0.48 to 0.89)**	**0.006**
Every day	0.81 (0.58 to 1.14)	0.226		0.88 (0.62 to 1.24)	0.456		0.82 (0.59 to 1.16)	0.264		0.86 (0.61 to 1.21)	0.378
Reported comorbidities											
**Diabetes mellitus**	**5.01 (3.69 to 6.80)**	**< 0.001**		**4.29 (3.12 to 5.89)**	**< 0.001**		**4.57 (3.35 to 6.22)**	**< 0.001**		**4.50 (3.30 to 6.15)**	**< 0.001**
Malignancy	0.81 (0.54 to 1.21)	0.297		0.74 (0.49 to 1.12)	0.151		0.78 (0.52 to 1.18)	0.239		0.75 (0.50 to 1.14)	0.178
Renal disease	1.62 (0.91 to 2.89)	0.104		1.40 (0.78 to 2.54)	0.261		1.66 (0.93 to 2.97)	0.088		1.53 (0.85 to 2.75)	0.154
Stroke	0.92 (0.39 to 2.16)	0.847		0.80 (0.33 to 1.91)	0.610		0.89 (0.38 to 2.10)	0.797		0.79 (0.33 to 1.89)	0.596
Congestive heart failure	0.41 (0.08 to 1.98)	0.266		0.44 (0.09 to 2.19)	0.319		0.39 (0.08 to 1.88)	0.240		0.39 (0.08 to 1.95)	0.254
Chronic lung disease	0.32 (0.10 to 1.02)	0.054		0.46 (0.14 to 1.48)	0.195		0.34 (0.11 to 1.09)	0.070		0.49 (0.15 to 1.52)	0.215
Eating disorder	0.81 (0.34 to 1.94)	0.634		0.61 (0.25 to 1.51)	0.287		0.77 (0.33 to 1.83)	0.556		0.62 (0.25 to 1.52)	0.296
**Depression**	**2.70 (1.82 to 4.01)**	**< 0.001**		**2.59 (1.72 to 3.90)**	**< 0.001**		**2.52 (1.69 to 3.75)**	**< 0.001**		**2.45 (1.64 to 3.66)**	**< 0.001**
**Other mental disorder**	**2.06 (1.33 to 3.20)**	**0.001**		**2.01 (1.26 to 3.19)**	**0.003**		**2.02 (1.30 to 3.13)**	**0.002**		**1.93 (1.23 to 3.04)**	**0.004**
Pseudo R^2^	0.1375			0.1813			0.1502			0.1604	

^1^Logistic regression model fitted with the inclusion of all variables listed above

The model without eating behavior evaluated Path C (the association between grit [exposure] and obesity [outcome]). The three models including eating behavior informally evaluated Path B (the association between eating behavior [mediator] and obesity [outcome]) and Path C’ (the association between grit [exposure] and obesity [outcome] after adjusting for eating behavior [mediator])

### Mediation effect of eating behavior on the relation between grit and obesity

The results of the mediation analyses are demonstrated in Table 4. The inverse association between grit and obesity was statistically accounted for by UE (96.5%) and EE (52.2%), consistent with the possibility of full mediation. In contrast, CR appeared to suppress the association, with an indirect effect of − 14.8%, suggesting that the direct effect exceeded the total effect. Sensitivity analyses that included regular exercise, smoking, and alcohol consumption as additional mediators showed similar patterns of statistical mediation (Additional File 10).

**Table 4:** Decomposition of the association between grit and obesity into direct and indirect effects using the KHB method^1^ (*N* = 1641)

	Eating behavior as a mediator							
	Uncontrolled eating			Cognitive restraint			Emotional eating	
	Odds ratio, point estimate (95% CI)	*P*		Odds ratio, point estimate (95% CI)	*P*		Odds ratio, point estimate (95% CI)	*P*
Indirect effect	**0.78 (0.72 to 0.84)**	**< 0.001**		**1.04 (1.01 to 1.07)**	**0.011**		**0.87 (0.83 to 0.92)**	**< 0.001**
Direct effect	0.99 (0.79 to 1.24)	0.937		**0.73 (0.59 to 0.91)**	**0.005**		0.88 (0.71 to 1.10)	0.263
Total effect	**0.77 (0.62 to 0.96)**	**0.021**		**0.76 (0.62 to 0.95)**	**0.013**		**0.77 (0.62 to 0.96)**	**0.018**
% of total effect mediated	96.5%			-14.8%			52.2%	

^1^The KHB method used is derived from a linear latent variable model assumed to underlie the logit model and extend the decomposition properties of the linear model to the logit model. This allowed the estimation of the overall, direct, and indirect effects in the logit model (obesity as the dependent variable, grit as the exposure variable, eating behavior as the mediator variable, and the other variables in Table 1 as covariates). This model formally evaluated Path C (association between grit and obesity), Path B (association between eating behavior and obesity), and Path C’ (association between grit and obesity after controlling for eating behavior)

## Discussion

Grit was positively associated with cognitive restraint and negatively associated with uncontrolled and emotional eating in the participants of this study. Consequently, the relation between grit and a lower likelihood of obesity was fully mediated by uncontrolled and emotional eating behaviors. However, the relation was only partially mediated by cognitive restraint, suggesting a potential direct association between grit and obesity that is not fully explained by the eating behavior pathway. Further research should focus on the abnormalities and causes of eating behaviors rather than ascribing the responsibility of obesity to an individual’s lack of willpower.

Our findings on the interrelations between grit, eating behaviors, and obesity align with previous neuroscientific research on grit and eating behavior, while extending prior work on grit and obesity. First, studies in the US that demonstrated correlations between grit and low BMI or a lower likelihood of obesity were limited to young adults and did not consider the differences in eating behaviors or exercise habits [10, 11]. As a result, the behavioral mechanisms linking grit to body composition remained unclear. Indeed, our study demonstrated that regular exercise was inversely associated with obesity, independent of grit. Second, our results on multidimensional eating behaviors associated with grit support previous research linking grit to brain activity involved in self-regulation. Individuals with high grit levels are more likely to engage in maintaining a regular diet and healthy food choices [9]. They also have been reported to show greater functional connectivity density in the right dorsolateral prefrontal cortex (DLPFC) [33], a brain region implicated in regulating eating behaviors. This neural profile may explain why higher grit is associated with less uncontrolled and emotional eating and greater cognitive restraint, contributing to the establishment of healthy eating habits. Third, unlike previous studies [10, 11], we demonstrated the potential mediating role of multidimensional eating behaviors in the relation of grit to obesity. Specifically, the low likelihood of obesity due to high grit appeared to be largely explained by the inhibition of uncontrolled or emotional eating. Sensitivity analyses further supported these findings, showing a larger proportion of mediation by eating behavior than by regular exercise.

Our findings highlight the need for healthcare providers and policymakers to consider individual differences in grit and the potential mediating role of multidimensional eating behaviors when developing strategies to manage obesity. First, the full mediation by uncontrolled and emotional eating underscores the importance of addressing these behaviors, particularly among individuals with low grit. Thus, healthcare providers may need to develop measures to assess and foster self-regulation over time through repeated dialogue about their thoughts, feelings, and eating behaviors [6, 34]. Indeed, mindfulness and cognitive behavioral therapy may help reduce BMI by inhibiting uncontrolled and emotional eating [34]. In addition, educational interventions for healthcare professionals, such as face-to-face group workshops aimed at enhancing empathy and understanding of the multifactorial causes and controllability of obesity [35], would help shift the clinical focus to eating behavior. Such training could also include goal setting around behavioral change, rather than weight alone [36], as a shared focus between individuals and providers. Second, one should not be bound by incorrect stereotypes that associate obesity with lack of willpower [5, 37], but rather re-emphasize that, as Stunkard et al. pioneered long ago, obesity is a multifaceted and complex problem that includes biology and behavioral science [38]. The presence of a direct effect on obesity associated with low grit, shown separately from an indirect effect via cognitive restraint, may indicate low resistance to obesogenic social conditions not captured by cognitive restraint items. For example, calorie-dense foods and beverages are less expensive and more accessible than fresh fruits and vegetables [5]. Additionally, the food industry’s promotion of these calorie-dense foods drives their purchase [5]. Therefore, policies should aim to improve these environments rather than attributing obesity to individual-level responsibility. Additionally, the observed suppression effect of cognitive restraint (–14.8%) suggests that individuals with high grit, particularly those with strong consistency of interest, engage in rigid dietary control in response to social pressures or obesity-related stigma. While such restraint may be well-intentioned, it could increase psychological stress and potentially contribute to disordered eating, thereby partially offsetting the protective effect of grit on obesity. In addition, the observed direct association between low grit and obesity may be partly explained by unmeasured or undiagnosed mental health conditions such as depression or ADHD. For example, low “perseverance of effort,” one component of grit, has been associated with depressive symptoms [39], and obesity co-occurring with depression is often linked to emotional and binge eating behaviors [40]. In our study, diagnosed depression was positively associated with uncontrolled, emotional, and restrained eating and with a higher likelihood of obesity. However, our analysis adjusted for diagnosed depression, suggesting that the relation of grit with obesity is not solely due to this condition. Likewise, lower levels of “consistency of interest” or “perseverance of effort” may reflect core traits of ADHD [41], such as impulsivity, which can lead to difficulties in regulating eating impulses [42]. ADHD in adulthood has also been linked to increased obesity risk [43]. These findings raise the possibility that undiagnosed or residual symptoms of ADHD or depression confound the observed direct association between grit and obesity, beyond the cognitive restraint pathway. Thus, while grit appears to influence obesity indirectly through eating behaviors, mental health conditions must be considered as potential overlapping or confounding factors in interpreting this relation.

The present study has several strengths. First, the inclusion of Japanese adult patients of both sexes across a wide range of age groups ensures the generalizability of our findings. Second, formal mediation analysis allowed us to quantify how multidimensional eating behaviors mediate the association between grit and obesity.

Nevertheless, this study had several limitations. First, the possibility of reverse causality cannot be excluded due to the cross-sectional nature of the study. For example, a potential change in thoughts about eating behaviors due to obesity (e.g., a strong preoccupation with the need for restraint due to obesity) would undermine the validity of our mediation analyses. Moreover, among individuals with obesity, there may be a subgroup who, paradoxically and self-deprecatingly, perceive themselves as lacking “perseverance of effort” due to their inability to lose weight, which could also contribute to reverse causality. Second, as in other studies [10], height and weight were self-reported, which may have led to underestimation of BMI. However, self-reported BMI is known to be highly correlated with measured BMI (Spearman’s ρ > 0.9) and is considered acceptable for assessing associations in epidemiological studies [44]. In addition, although BMI ≥ 25 kg/m² is an appropriate threshold for defining obesity in the Japanese population, BMI does not directly reflect body fat content or distribution. For instance, an individual with high muscle mass may have elevated BMI despite low adiposity, while older adults with sarcopenia may have normal BMI but high body fat percentage. Third, whether the present findings, derived from a single-ethnic population within the Japanese food culture, apply to other countries or racial groups is uncertain. However, the observed mechanisms may be particularly relevant across cultures among individuals with low socioeconomic status, who often face barriers to healthy eating due to economic constraints and greater exposure to highly accessible, calorie-dense foods. In such contexts, perseverance toward healthy eating (e.g., grit) may be undermined by structural challenges, contributing to emotional or uncontrolled eating and increased obesity risk. The higher prevalence of obesity among low-income young Japanese women may support this notion [45].

## Conclusions

In conclusion, among Japanese adults across a wide age range, the inverse association between grit and obesity was statistically accounted for by differences in uncontrolled eating, emotional eating, and cognitive restraint. These findings are consistent with a potential mediating role of multidimensional eating behaviors in the association between grit and obesity. However, our findings also suggest a possible direct association of grit with obesity, independent of cognitive restraint. These results emphasize the complex interplay between personality traits and eating behaviors in shaping obesity risk. Rather than focusing solely on grit, addressing the underlying causes of uncontrolled and emotional eating may contribute to more effective obesity prevention strategies at both the individual and societal level.

## Supplementary Information

Below is the link to the electronic supplementary material.

Supplementary Material 1

Supplementary Material 2

Supplementary Material 3

Supplementary Material 4

Supplementary Material 5

Supplementary Material 6

Supplementary Material 7

Supplementary Material 8

Supplementary Material 9

Supplementary Material 10

## Data Availability

The datasets generated/analyzed during the current study are available from the corresponding author upon reasonable request.

## Abbreviations

BMI: Body mass index
EE: Emotional eating
TFEQ: Three-factor eating questionnaire
UE: Uncontrolled eating
CR: Cognitive restraint
TFEQ-R21: Japanese version of the 21-item three-factor eating questionnaire-R21
DEBQ: Dutch eating behavior questionnaire
SDs: Standard deviations
CFI: Comparative fit index
RMSEA: Root mean square approximation error
KHB: Karlson–-Holm–-Breen
aOR: Adjusted odds ratio
DLPFC: Dorsolateral prefrontal cortex
