# Supplementary material for: The grit personality trait, eating behavior, and obesity among Japanese adults: a cross-sectional study
Source: Biopsychosoc Med. 2025 Aug 22;19:15. doi: 10.1186/s13030-025-00337-9 (PMC12372174; doi:10.1186/s13030-025-00337-9)
Supplement: Supplementary file 1 — Supplementary Material 1 [file 13030_2025_337_MOESM1_ESM.docx]

# Additional File 1.

# A. Participant sampling strategy

Our panel survey recruited Japanese adults aged ≥ 20 years with the assistance of a web-based company (Cross Marketing, Shinjuku, Tokyo, Japan). The target sample size was 1500 participants due to the project's budget constraints. The sample size was set at a 1:1 ratio for men and women (750 each) and a 1:1 ratio for older and younger individuals of each sex (375 each for each sex). To sample individuals with and without obesity, sampling ratios were established using the original categories for which the web-based company panel was registered in advance. Specifically, we set the number of individuals to ensure a 10:3:2 ratio among those who reported a history of hospitalization or clinic visits for obesity, those who were worried about obesity, and those who did not report these problems (i.e., the general population) (i.e., 250, 75, and 50 individuals for each sex and each age group, respectively).

**Additional File 1.**

**B. Designing Screener Items**

To assess the presence of careless participants [[1]](https://paperpile.com/c/yiLwwt/rEPUf), five “screener” items were created to identify and exclude them from our analyses. Specifically, we excluded respondents with an inappropriate entry of 1) age, 2) sex or extreme values for 3) height or 4) weight, or those with 5) completion times < 5 min [[2, 3]](https://paperpile.com/c/yiLwwt/LGyDR+wMKeM).

The respondents were asked to indicate their age and sex twice, once at the beginning and once in the latter part of the questionnaire. We identified and excluded respondents whose age or sex responses did not match.

In the latter part of the questionnaire, the respondents were asked to indicate their height and weight. Considering the realistic distribution of anthropometric characteristics among Japanese individuals, we excluded those who reported their height to be either ≥ 210 cm or ≤ 120 cm. We also excluded those who reported their weight to be either ≥ 151 kg or ≤ 25 kg.

Based on our pilot test of the time required to complete the questionnaire, we excluded respondents who completed the survey in less than five minutes. Those who completed the questionnaire too quickly were categorized as careless [[1, 2]](https://paperpile.com/c/yiLwwt/rEPUf+LGyDR) and were excluded from our analyses.

**References**

1. Berinsky AJ, Margolis MF, Sances MW. Separating the shirkers from the workers? Making sure respondents pay attention on self‐administered surveys. Am J Pol Sci. 2014*;*58*:*739–53. <https://doi.org/10.1111/ajps.12081>

2. [Meade AW, Craig SB. Identifying careless responses in survey data. Psychol Methods*.* 2012*;*17*:*437–55. <https://doi.org/10.1037/a0028085>](http://paperpile.com/b/yiLwwt/LGyDR)

3. [Suzuki R, Yajima N, Sakurai K, Oguro N, Wakita T, Thom DH, et al. Association of patients’ past misdiagnosis experiences with trust in their current physician among Japanese adults. J Gen Intern Med. 2022*;*37*:*1115–21. <https://doi.org/10.1007/s11606-021-06950-y>](http://paperpile.com/b/yiLwwt/wMKeM)
