# Supplementary material for: The grit personality trait, eating behavior, and obesity among Japanese adults: a cross-sectional study
Source: Biopsychosoc Med. 2025 Aug 22;19:15. doi: 10.1186/s13030-025-00337-9 (PMC12372174; doi:10.1186/s13030-025-00337-9)
Supplement: Supplementary file 2 — Supplementary Material 2 [file 13030_2025_337_MOESM2_ESM.docx]

**Additional File 2. Japanese version of The Three-Factor Eating Questionnaire-Revised 21-Item (TFEQ-R21)**

| Instruction | 以下の文章について、あなたはどの程度そう思いますか。最もよく表している回答の番号に○をつけてください。*  (Original: “Please circle the number for the response that best describes how much you agree or disagree with each of the following statements.”) |
| --- | --- |
| Question 1 | 体重をコントロールするため、盛り付けやとりわけは意識して少なくしている。†  (Original: “I deliberately take small helpings to control my weight.”) |
| Question 2 | 不安を感じると食べ始めてしまう。†  (Original: “I start to eat when I feel anxious.”) |
| Question 3 | 食べ始めると、どうにも止められなくなることがある。†  (Original: “Sometimes when I start eating, I just can’t seem to stop.”) |
| Question 4 | 悲しくなると、食べ過ぎてしまうことがよくある。†  (Original: “When I feel sad, I often eat too much.”) |
| Question 5 | 食べると太るという理由で食べない食品がある。†  (Original: “I don’t eat some foods because they make me fat.”) |
| Question 6 | 一緒にいる人が食べていると、自分も食べたくなることがよくある。†  (Original: “Being with someone who is eating often makes me want to also eat.”) |
| Question 7 | 気持ちが張り詰めたり、"いっぱいっぱい"になったりすると、食べたいと強く思うことがよくある。†  (Original: “When I feel tense or ‘‘wound up’’, I often feel I need to eat.”) |
| Question 8 | お腹が減りすぎて、胃が底なしに感じることがよくある。†  (Original: “I often get so hungry that my stomach feels like a bottomless pit.”) |
| Question 9 | 途中で止められずにお皿の上の食品を全て食べてしまうほど、いつもお腹が空いている。†  (Original: “My doctor is well qualified to manage (diagnose and treat or make an appropriate referral) medical problems like mine.”) |
| Question 10 | 孤独を感じると、食べて自分をなぐさめる。†  (Original: “When I feel lonely, I console myself by eating.”) |
| Question 11 | 体重が増えないように、意識して食事の量を抑えるようにしている。†  (Original: “I consciously hold back on how much I eat at meals to keep from gaining weight.”) |
| Question 12 | 自分の好物が調理されているにおいをかいだり見たりすると、たとえ食事したばかりであっても、食べずにはいられない。†  (Original: “When I smell a sizzling steak or see a juicy piece of meat, I find it very difficult to keep from eating - even if I've just finished a meal.”) |
| Question 13 | いつでも食べられるくらい、つねにお腹が空いている。†  (Original: “ I’m always hungry enough to eat at any time.”) |
| Question 14 | 緊張すると、食べて落ち着こうとする。†  (Original: “If I feel nervous, I try to calm down by eating.”) |
| Question 15 | とてもおいしそうなものを目にしたら、すぐ食べずにいられないぐらい、おなかがすくことがよくある。†  (Original: “When I see something that looks very delicious, I often get so hungry that I have to eat right away.”) |
| Question 16 | 気持ちが落ち込んだときは、食べたくなる。†  (Original: “When I feel depressed, I want to eat.”) |
| Response options for Questions 1 to 16 | 全く当てはまる/ほぼ当てはまる/ほぼ当てはまらない/ 全く当てはまらない  (Original: Definitely true/Mostly true/Mostly false/Definitely false) |
| Question 17 | 誘惑に負けそうな食べ物の「買いおき」をどのくらい避けていますか？  (Original: “How often do you avoid ‘‘stocking up’’ on tempting foods?”) |
| Response options for Question 17 | ほぼ避けない/たまにしか避けない/ 通常は避ける/ほぼいつも避ける  (Original: Almost never/Seldom/Usually/Almost always) |
| Question 18 | 自分が食べたいと思う量よりも食べる量を少なくしようとすることはどのくらい ありそうですか？  (Original: “How likely are you to make an effort to eat less than you want?”) |
| Response options for Question 18 | あまりない/少しある/ まぁまぁある/大いにある  (Original: Unlikely/A little likely/Somewhat likely/Very likely) |
| Question 19 | お腹がすいていないのに、一気に極端な量を食べてしまうことはありますか？  (Original: “Do you go on eating binges even though you’re not hungry?”) |
| Response options for Question 19 | まったくない/めったにない/ ときどきある/週に1回以上ある (Original: Never/Rarely/Sometimes/At least once a week) |
| Question 20 | お腹が空くと感じることがどのくらいありますか？  (Original: “How often do you feel hungry?”) |
| Response options for Question 20 | 食事の時だけ感じる/食事の合間もときどき感じる/ 食事の合間もよく感じる/ほとんどいつも感じる  (Original: Only at mealtimes/Sometimes between meals/Often between meals/Almost always) |
| Question 21 | 食事を全く制限していない状態を1、完全に制限している状態を8として1から8で あらわしたときに、自分はいくつだと思いますか？ 最も自分にあてはまる数字に〇をつけてください。‡  (Original: “On a scale from 1 to 8, where 1 means no restraint in eating and 8 means total restraint, what number would you give yourself? Mark the number that best applies to you”) |
| Response options for Question 21 | \|  \| 1 \| 2 \| 3 \| 4 \| 5 \| 6 \| 7 \| 8 \|  \| \| --- \| --- \| --- \| --- \| --- \| --- \| --- \| --- \| --- \| --- \|   常に自分の食事摂取を制限しており、決してゆずらない  いつでも食べたいときに、なんでも食べたいものを食べる       \|  \| 1 \| 2 \| 3 \| 4 \| 5 \| 6 \| 7 \| 8 \|  \| \| --- \| --- \| --- \| --- \| --- \| --- \| --- \| --- \| --- \| --- \|   I eat whatever  and whenever  I want to  I am constantly limiting my food intake, never "giving in" |

The original English version [[1]](https://paperpile.com/c/yiLwwt/WYRSU) is also provided for each item and response.

*The instructional statements generated through the formal process of translation are presented.

†Questions 1 through Question 16 are reverse-scored items.

‡Question 21 was re-coded as follows: scores 1–2 as 1, 3–4 as 2, 5–6 as 3, and 7–8 as 4.

Using the twenty-one questions, the following three domains can be assessed:

**Uncontrolled eating (UE)**: Question 3, Question 6, Question 8, Question 9, Question 12, Question 13, Question 15, Question 19, and Question 20.

**Cognitive restraint (CR)**: Question 1, Question 5, Question 11, Question 17, Question 18, and Question 21.

**Emotional eating (EE)**: Question 2, Question 4, Question 7, Question 10, Question 14, and Question 16

Before using this instrument, please register at https://noriaki-kurita.jp/resources/tfeq-r21-jpn/.

In addition, please cite this article as follows:

Kurita N, Maeshibu T, Aita T, Wakita T, Kikuchi H. The grit personality trait, eating behavior, and obesity among Japanese adults: a cross-sectional study. *BioPsychoSocial Medicine*. 2025. DOI: 10.1186/s13030-025-00337-9

**Reference**

1**.** [Cappelleri JC, Bushmakin AG, Gerber RA, Leidy NK, Sexton CC, Lowe MR, et al. Psychometric analysis of the Three-Factor Eating Questionnaire-R21: results from a large diverse sample of obese and non-obese participants. Int J Obes (Lond). 2009*;*33*:*611–20. <https://doi.org/10.1038/ijo.2009.74>](http://paperpile.com/b/yiLwwt/WYRSU)
