# Supplementary material for: The grit personality trait, eating behavior, and obesity among Japanese adults: a cross-sectional study
Source: Biopsychosoc Med. 2025 Aug 22;19:15. doi: 10.1186/s13030-025-00337-9 (PMC12372174; doi:10.1186/s13030-025-00337-9)
Supplement: Supplementary file 3 — Supplementary Material 3 [file 13030_2025_337_MOESM3_ESM.docx]

**Additional File 3. Survey items used for analyses in the present study**

The Japanese version of the Dutch Eating Behavior Questionnaire (DEBQ) is a 33-item instrument consisting of three factors: “emotional eating,” which indicates eating behavior aroused by heightened emotions (13 items); “external eating,” implying eating behavior triggered by external stimuli such as taste and smell (10 items); and, “restrained eating,” implying the degree of intentional dietary restriction (10 items) [[1, 2]](https://paperpile.com/c/yiLwwt/qxkoe+eUMPI). The respondents were instructed to rate each item on a Likert scale ranging from 1 (never) to 5 (very often). The average of the items was used as the score for each of the three domains [[1]](https://paperpile.com/c/yiLwwt/qxkoe). Higher scores indicated that the construct was applied better. The alpha coefficients for each domain were as follows: emotional eating, 0.95; external eating, 0.73; and restrained eating, 0.87 [[2]](https://paperpile.com/c/yiLwwt/eUMPI). Criterion-related validity of the Japanese version of the DEBQ was tested against the body mass index in Japanese adults; although a weak negative correlation with external eating and a weak positive correlation with restrained eating was demonstrated, a correlation with emotional eating was not demonstrated [[2]](https://paperpile.com/c/yiLwwt/eUMPI).

Demographic characteristics such as age, sex, education level, total household income, and marital status; healthy behaviors such as exercise habits, smoking history, and alcohol consumption; and non-communicable diseases, such as diabetes, cancer, kidney disease, stroke, congestive heart failure, chronic lung disease, eating disorders, depression, and other mental disorders were collected as covariates.

Sex was selected as either male or female. The educational level was junior high school, high school, professional training college, technology college, junior college, university, or graduate school. For the analyses, the data were collapsed into five levels (junior high school, high school, professional training college/college of technology/junior college, university, and graduate school). Total household income was classified as < 1 000 000 yen; 1 000 000 to < 2 999 999 yen; 3 000 000 to < 4 999 999 yen; 5 000 000 to < 9 999 999 yen; ≥ and 10,000,000 yen. For the analyses, the data were collapsed into four levels < 1 000 000 yen; 1 000 000 to < 2 999 999 yen; 3 000 000 to < 4 999 999 yen; 5 000 000 to < 9 999 999 yen; ≥ and 10,000,000 yen). Marital status was classified as unmarried, married, divorced, or widowed. The items on exercise habits, smoking, and alcohol consumption were adapted from a standard questionnaire for specific health checkups among Japanese adults as suggested by the Ministry of Health, Labor, and Welfare, Japan [[3]](https://paperpile.com/c/yiLwwt/adzLH). Regular exercise was defined if the respondent chose “yes” from “yes” or “no” to the following question: “I do light, sweaty exercise of at least 30 minutes two days a week for at least one year.” This question corresponds to ≥ 60 minutes per week of physical activity with an intensity ≥ 3 METS, which has been shown to reduce the risk of lifestyle-related diseases and mortality by 12% [[3]](https://paperpile.com/c/yiLwwt/adzLH). Smoking was defined by a response of “yes” from “yes” or “no” to the following question: “Do you currently habitually smoke cigarettes?” The question was annotated so that “current habitual smokers” were defined as “those who smoked a total of ≥100 cigarettes or smoked for ≥6 months” and those who smoked during the last month. Alcohol consumption was chosen from one of the following three options in response to the question: “Frequency of alcohol consumption (sake, shochu, beer, western-style liquor, etc.)”: every day, sometimes, rarely or never (including inability to drink). For noncommunicable diseases, the following instructional statement was used: “Have you ever been told by a physician that you have any of the following diseases?” The respondents selected from the following nine items: diabetes mellitus, cancer, kidney disease, stroke, congestive heart failure, chronic lung disease, eating disorders, depression, and other mental disorders. For each item, one of three options was allowed: never told, told, and visited a physician in the past, told and currently visiting a physician's office. If either of the latter two were chosen, the respondent was considered to have a non-communicable disease.

**References**

1. [van Strien T, Frijters JER, Bergers GPA, Defares PB. The Dutch Eating Behavior Questionnaire (DEBQ) for assessment of restrained, emotional, and external eating behavior. Int J Eat Disord. 1986*;*5*:*295–315. [https://doi.org/10.1002/1098-108X(198602)5:2<295::AID-EAT2260050209>3.0.CO;2-T](https://doi.org/10.1002/1098-108x(198602)5:2%3c295::aid-eat2260050209%3e3.0.co;2-t)](http://paperpile.com/b/yiLwwt/qxkoe)

2. [Takayama N, Amemiya T, Nishikawa K, Yoshizu J, Ariyoshi H, Suzaki Y, et al. Research on eating behavior of adult workers and adolescent students using Dutch Eating Behavior Questionnaire. J Jpn Health Med Assoc*.* 2012*;*21*:*87–94](http://paperpile.com/b/yiLwwt/eUMPI)

3. [Ministry of Health, Labour and Welfare. Questionnaire of specific health checkups. Secondary questionnaire of specific health checkups.](http://paperpile.com/b/yiLwwt/adzLH) <https://www.mhlw.go.jp/seisakunitsuite/bunya/kenkou_iryou/kenkou/seikatsu/dl/hoken-program2_02.pdf>. Accessed 19 Dec 2023
