# Supplementary material for: The grit personality trait, eating behavior, and obesity among Japanese adults: a cross-sectional study
Source: Biopsychosoc Med. 2025 Aug 22;19:15. doi: 10.1186/s13030-025-00337-9 (PMC12372174; doi:10.1186/s13030-025-00337-9)
Supplement: Supplementary file 5 — Supplementary Material 5 [file 13030_2025_337_MOESM5_ESM.docx]

**Additional File 5**

**Three-factor structural validity and internal consistency reliability of the domains in the TFEQ-R21**

| **Goodness-of-fit indices based on confirmatory factor analysis** |  |
| --- | --- |
| Comparative Fit Index | 0.924 |
| Root Mean Square Error of Approximation | 0.069 |
| Standardized Root Mean Square Residual | 0.059 |
|  |  |
| **Internal consistency reliability** |  |
| Cronbach's α coefficient |  |
| Uncontrolled Eating | 0.89 |
| Cognitive Restraint | 0.79 |
| Emotional Eating | 0.92 |
|  |  |
| McDonald's ω coefficient |  |
| Uncontrolled Eating | 0.89 |
| Cognitive Restraint | 0.78 |
| Emotional Eating | 0.93 |
|  |  |
