# Supplementary material for: The grit personality trait, eating behavior, and obesity among Japanese adults: a cross-sectional study
Source: Biopsychosoc Med. 2025 Aug 22;19:15. doi: 10.1186/s13030-025-00337-9 (PMC12372174; doi:10.1186/s13030-025-00337-9)
Supplement: Supplementary file 6 — Supplementary Material 6 [file 13030_2025_337_MOESM6_ESM.docx]

# Additional File 6. Confirmatory factor analysis for TFEQ-R21

Dark grey squares indicate the items. Dark grey circles indicate domains. Digits overlaid on the one-way arrows indicate standardized loadings. The digits overlaid on the two-way arrows indicate the correlations.
