# Supplementary material for: The grit personality trait, eating behavior, and obesity among Japanese adults: a cross-sectional study
Source: Biopsychosoc Med. 2025 Aug 22;19:15. doi: 10.1186/s13030-025-00337-9 (PMC12372174; doi:10.1186/s13030-025-00337-9)
Supplement: Supplementary file 8 — Supplementary Material 8 [file 13030_2025_337_MOESM8_ESM.docx]

**Additional File 8. Criterion validity of the TFEQ-R21**

|  | DEBQ | | |  |
| --- | --- | --- | --- | --- |
|  | External eating | Restrained eating | Emotional eating | BMI |
| TFEQ-R21 |  |  |  |  |
| Uncontrolled eating | 0.6605; p <0.001 | 0.1155; p <0.001 | 0.6706; p <0.001 | 0.3173; p <0.001 |
| Cognitive restraint | 0.0420; p =0.0889 | 0.7572; p <0.001 | 0.1610; p <0.001 | 0.2080; p <0.001 |
| Emotional eating | 0.5050; p <0.001 | 0.1433; p <0.001 | 0.8029; p <0.001 | 0.2889; p <0.001 |

Pearson correlation coefficients between the three factors in the TFEQ-R21 and the three factors in the DBEQ and BMI are shown.

TFEQ-R21, The 21-item Three-Factor Eating Questionnaire; DEBQ, Dutch Eating Behavior Questionnaire; BMI, body mass index.
