# Supplementary material for: The grit personality trait, eating behavior, and obesity among Japanese adults: a cross-sectional study
Source: Biopsychosoc Med. 2025 Aug 22;19:15. doi: 10.1186/s13030-025-00337-9 (PMC12372174; doi:10.1186/s13030-025-00337-9)
Supplement: Supplementary file 9 — Supplementary Material 9 [file 13030_2025_337_MOESM9_ESM.docx]

**Additional File 9. Correlation matrix of the TFEQ-R21**

|  | TFEQ-R21 | | |
| --- | --- | --- | --- |
|  | Uncontrolled eating | Cognitive restraint | Emotional eating |
| TFEQ-R21 |  |  |  |
| Uncontrolled eating | 1.000 |  |  |
| Cognitive restraint | 0.1684; p <0.001 | 1.000 |  |
| Emotional eating | 0.7812; p <0.001 | 0.2161; p <0.001 | 1.000 |

Pearson correlation coefficients among the three domains of the TFEQ-R21 are shown.

TFEQ-R21, The 21-item Three-Factor Eating Questionnaire.
