# Supplementary material for: The grit personality trait, eating behavior, and obesity among Japanese adults: a cross-sectional study
Source: Biopsychosoc Med. 2025 Aug 22;19:15. doi: 10.1186/s13030-025-00337-9 (PMC12372174; doi:10.1186/s13030-025-00337-9)
Supplement: Supplementary file 10 — Supplementary Material 10 [file 13030_2025_337_MOESM10_ESM.docx]

# Additional File 10. Decomposition of the association between grit and obesity into direct and indirect effects using the KHB method† (N = 1641)

|  | **Eating behavior as one of multiple mediators** | | | | | | | |
| --- | --- | --- | --- | --- | --- | --- | --- | --- |
|  | **Uncontrolled eating** |  |  | **Cognitive restraint** |  |  | **Emotional eating** |  |
|  | Odds ratio, point estimate (95% CI) | P |  | Odds ratio, point estimate (95% CI) | P |  | Odds ratio, point estimate (95% CI) | P |
| Indirect effect |  |  |  |  |  |  |  |  |
| *Overall* | **0.75 (0.69 to 0.81)** | **< .001** |  | 1.002 (0.95 to 1.05) | .938 |  | **0.84 (0.78 to 0.9)** | **< .001** |
|  |  |  |  |  |  |  |  |  |
| *Eating behavior* | **0.78 (0.73 to 0.84)** |  |  | **1.05 (1.01 to 1.08)** |  |  | **0.87 (0.83 to 0.92)** |  |
| *Regular exercise* | **0.96 (0.92 to 0.99)** |  |  | **0.96 (0.93 to 0.99)** |  |  | **0.96 (0.93 to 0.992)** |  |
| *Smoking* | 1.001 (0.994 to 1.007) |  |  | 1 (0.998 to 1.003) |  |  | 1 (0.996 to 1.005) |  |
| *Alcohol consumption* |  |  |  |  |  |  |  |  |
| *Rarely or never (including inability to drink)* | Ref. |  |  | Ref. |  |  | Ref. |  |
| *Sometimes* | 0.99 (0.97 to 1.008) |  |  | 0.99 (0.97 to 1.008) |  |  | 0.99 (0.97 to 1.008) |  |
| *Every day* | 1.01 (0.99 to 1.02) |  |  | 1.01 (0.993 to 1.02) |  |  | 1.01 (0.99 to 1.02) |  |
|  |  |  |  |  |  |  |  |  |
| Direct effect | 0.99 (0.79 to 1.24) | .937 |  | **0.73 (0.59 to 0.91)** | **.005** |  | 0.88 (0.71 to 1.1) | .263 |
| Total effect | **0.74 (0.6 to 0.92)** | **.007** |  | **0.73 (0.59 to 0.91)** | **.004** |  | **0.74 (0.6 to 0.92)** | **.006** |
|  |  |  |  |  |  |  |  |  |
| % of Total Effect mediated |  |  |  |  |  |  |  |  |
| *Overall* | 97% |  |  | -0.6% |  |  | 58.6% |  |
|  |  |  |  |  |  |  |  |  |
| *Eating behavior* | 80.7% |  |  | -14.4% |  |  | 44.5% |  |
| *Regular exercise* | 15.1% |  |  | 13.4% |  |  | 13.3% |  |
| *Smoking* | -0.2% |  |  | -0.1% |  |  | -0.1% |  |
| *Alcohol consumption* |  |  |  |  |  |  |  |  |
| *Rarely or never (including inability to drink)* | — |  |  | — |  |  | — |  |
| *Sometimes* | 3.1% |  |  | 3.1% |  |  | 3.1% |  |
| *Every day* | -1.8% |  |  | -2.6% |  |  | -2.1% |  |

^†^The KHB method was used, which is derived from a linear latent variable model assumed to underlie the logit model and extend the decomposition properties of the linear model to the logit model. This allowed for the estimation of the overall, direct, and indirect effects in the logit model (obesity as the dependent variable; grit as the exposure variable; eating behavior, regular exercise, smoking, and alcohol as the mediator variables; and the other variables in Table 1 as covariates). KHB, Karlson–-Holm–-Breen.
